# Powered Hip Exoskeleton Reduces Residual Hip Effort Without Affecting Kinematics and Balance in Individuals With Above-Knee Amputations During Walking

**DOI:** 10.1109/TBME.2022.3211842

**Published:** 2023-03-21

**Authors:** Marshall K. Ishmael, Andrew Gunnell, Kai Pruyn, Suzi Creveling, Grace Hunt, Sarah Hood, Dante Archangeli, K. Bo Foreman, Tommaso Lenzi

**Affiliations:** Department of Mechanical Engineering, Robotics Center, University of Utah, USA.; Department of Mechanical Engineering, Robotics Center, University of Utah, USA.; Department of Mechanical Engineering, Robotics Center, University of Utah, USA; Department of Biomedical Engineering, University of Utah, USA.; Department of Mechanical Engineering, Robotics Center, University of Utah, USA.; Department of Mechanical Engineering, Robotics Center, University of Utah, USA.; Department of Mechanical Engineering, Robotics Center, University of Utah, USA.; Department of Mechanical Engineering, Robotics Center, University of Utah, USA.; Department of Mechanical Engineering, Robotics Center, University of Utah, USA; Department of Physical Therapy and Athletic Training, University of Utah, USA.; Department of Mechanical Engineering, Robotics Center, University of Utah, Salt Lake City, UT 84112 USA

**Keywords:** Assistive/rehabilitation robotics, biomechanics, biomimetic & bio-inspired robotics, powered hip exoskeleton, prosthetics, robotics, transfemoral amputation

## Abstract

**Objective:**

A unilateral, lightweight powered hip exoskeleton has been shown to improve walking economy in individuals with above-knee amputations. However, the mechanism responsible for this improvement is unknown. In this study we assess the biomechanics of individuals with above-knee amputations walking with and without a unilateral, lightweight powered hip exoskeleton. We hypothesize that assisting the residual limb will reduce the net residual hip energy.

**Methods:**

Eight individuals with above-knee amputations walked on a treadmill at 1 m/s with and without a unilateral powered hip exoskeleton. Flexion/extension assistance was provided to the residual hip. Motion capture and inverse dynamic analysis were performed to assess gait kinematics, kinetics, center of mass, and center of pressure.

**Results:**

The net energy at the residual hip decreased from 0.05±0.04 J/kg without the exoskeleton to *−*0.01±0.05 J/kg with the exoskeleton (p = 0.026). The cumulative positive energy of the residual hip decreased on average by 18.2% with 95% confidence intervals (CI) (0.20 J/kg, 0.24 J/kg) and (0.16 J/kg, 0.20 J/kg) without and with the exoskeleton, respectively. During stance, the hip extension torque of the residual limb decreased on average by 37.5%, 95% CI (0.28 Nm/kg, 0.36 Nm/kg), (0.17 Nm/kg, 0.23 Nm/kg) without and with the exoskeleton, respectively.

**Conclusion:**

Powered hip exoskeleton assistance significantly reduced the net residual hip energy, with concentric energy being the main contributor to this change. We believe that the reduction in residual hip extension torque during early stance is the main contributor to this reduction.

**Significance:**

This analysis shows that by assisting the residual hip, the exoskeleton significantly decreased the net hip energy produced by the residual limb, which may explain the improvements in walking economy previously observed.

## I. Introduction

Conventional knee and ankle prostheses are energetically passive devices that cannot provide biomechanically accurate torque, power, and movements during ambulation [1]. Individuals with above-knee amputations compensate for the limited functionality of their passive prostheses with complex compensatory strategies involving their sound leg, their residual limb above the amputation level, their pelvis, and their upper body [2], [3]. Clinical research has shown that these compensatory gait strategies have negative effects on walking balance, stability, speed, and efficiency [4], limiting the movement ability and quality of life of individuals with leg amputations. Moreover, the compensatory gait strategies used with lower-limb prostheses have been frequently linked to debilitating secondary health issues such as osteoarthritis [5] and lower-back pain [6]. These studies motivate the development of new assistive technologies and interventions for individuals with lower-limb amputations.

Clinical gait analysis highlights the functional limitations of existing prostheses and the compensatory strategies used by individuals with above-knee amputations during walking. Inverse dynamics analysis of nonamputee gait shows that the biological ankle provides net-positive energy [7], [8]. However, most conventional prostheses are passive. Therefore, they cannot provide energy injection, which is a critical function of biological legs. Individuals with above-knee amputations compensate for the loss of biological ankle energy on the prosthesis side using different strategies. One strategy is to increase the effort in their residual, prosthesis side hip [9]. This residual hip compensation strategy is particularly hard for individuals with above-knee amputations because the residual limb muscles are rearranged during amputation surgery in a non-physiological manner [10], which reduces the strength and range of motion of the residual hip joint [11]. Individuals with above-knee amputations also compensate for the lack of energy from their prostheses by increasing the effort in their sound hip and ankle [12]. Due to these compensatory movements, the pelvic motion increases by a factor of two [13], [14]. Although these compensatory movements are observed in many subjects [15], trunk lean and atrophied hip muscles due to amputation surgery may actually result in reduced prosthesis side hip effort [16]. Moreover, knee prosthesis selection has a noticeable effect on hip kinematics [17] and walking stability [18]. Similarly, socket selection and the residual limb length affect the kinematics of the residual hip joint [13]. These studies provide a mechanistic explanation for the asymmetric gait pattern observed in individuals with above-knee amputations, motivating the development of more advanced assistive technologies that can overcome the fundamental limitations of existing leg prostheses [19], [20].

Powered prostheses have been proposed to more closely replicate the biomechanical function of the missing biological leg. For example, powered prostheses can inject net-positive energy into the gait cycle with battery-powered servomotors which can imitate the energy-injection function of the biological ankle [21], [22]. Unfortunately, existing powered prostheses are substantially heavier than their passive counterparts. Biomechanical studies have shown that additional mass placed on body segments increases the effort required to walk, and that the additional energetic exertion is proportional to the distance of the additional mass from the whole-body center of mass. For example, adding mass at the ankle increases metabolic cost four times more than at the trunk [23]. Moreover, increased prosthesis mass correlates with increased energy expenditure and increased gait asymmetry [24]. For example, matching the prosthesis weight to the weight of the missing biological limb resulted in a 12% increase in the metabolic cost of walking [25], and increasing the inertia of the prosthesis exacerbated stance and swing time asymmetries [26]. Despite recent efforts [27], [28], reducing the weight of powered prostheses without deterio-rating performance is an open challenge. Therefore, the potential benefits of powered prostheses are offset by the increased distal mass of these devices.

An alternative approach to improve amputee walking economy is assisting the residual or sound hips with a motorized orthosis or a powered hip exoskeleton [29], [30], [31], [32], [33]. A powered hip exoskeleton can be made lightweight because the onboard motors only need to provide a fraction of the torque generated by the biological hip. Moreover, the mass of a powered hip exoskeleton is located close to the trunk, minimizing the negative effects on user’s gait mechanics and energetics [23]. Perhaps because of their small and proximal mass, powered hip exoskeletons have recently succeeded in significantly reducing the metabolic cost of walking in individuals with above-knee amputations [29], [33], which has never been shown with powered prostheses. Powered hip exoskeletons have also been effective gait training devices [30] and stumble recovery aids in individuals with above-knee amputations [31]. These positive clinical outcomes suggest that powered hip exoskeletons are a viable tool to improve gait in individuals with above-knee amputations. However, the effect of the assistance provided by a hip exoskeleton on amputee gait mechanics is still unknown. Thus, there is no mechanistic explanation for the improved metabolic cost of walking that has been shown with powered hip exoskeletons.

In this paper, we assess the effect of residual limb assistance provided by a unilateral, lightweight powered hip exoskeleton on gait kinematics and kinetics of individuals with above-knee amputations. We hypothesize that assisting the residual limb with a unilateral, lightweight powered hip exoskeleton will decrease the torque generated by the residual limb muscles at the hip joint, reducing the residual limb energy. We will test this hypothesis by performing motion capture and inverse dynamic analysis while eight participants with unilateral above-knee amputations walk with and without an autonomous, lightweight powered hip exoskeleton assisting their residual limb. We will use net hip energy at the residual limb as the primary outcome. We will also perform secondary analyses on kinematics, gait timing, balance, and stability to assess the effect of asymmetric assistance on compensatory behaviors, as well as to assess changes associated with wearing the assistive exoskeleton.

By providing new insights into how above-knee amputees adapt their biomechanics to exoskeleton assistance, this paper contributes new knowledge related to the residual hip-assistance approach. This knowledge may suggest new ways to improve clinical outcomes for individuals with above-knee amputations.

## II. Methods

### A. Unilateral, Lightweight Powered Hip Exoskeleton

**Fig. 1.**
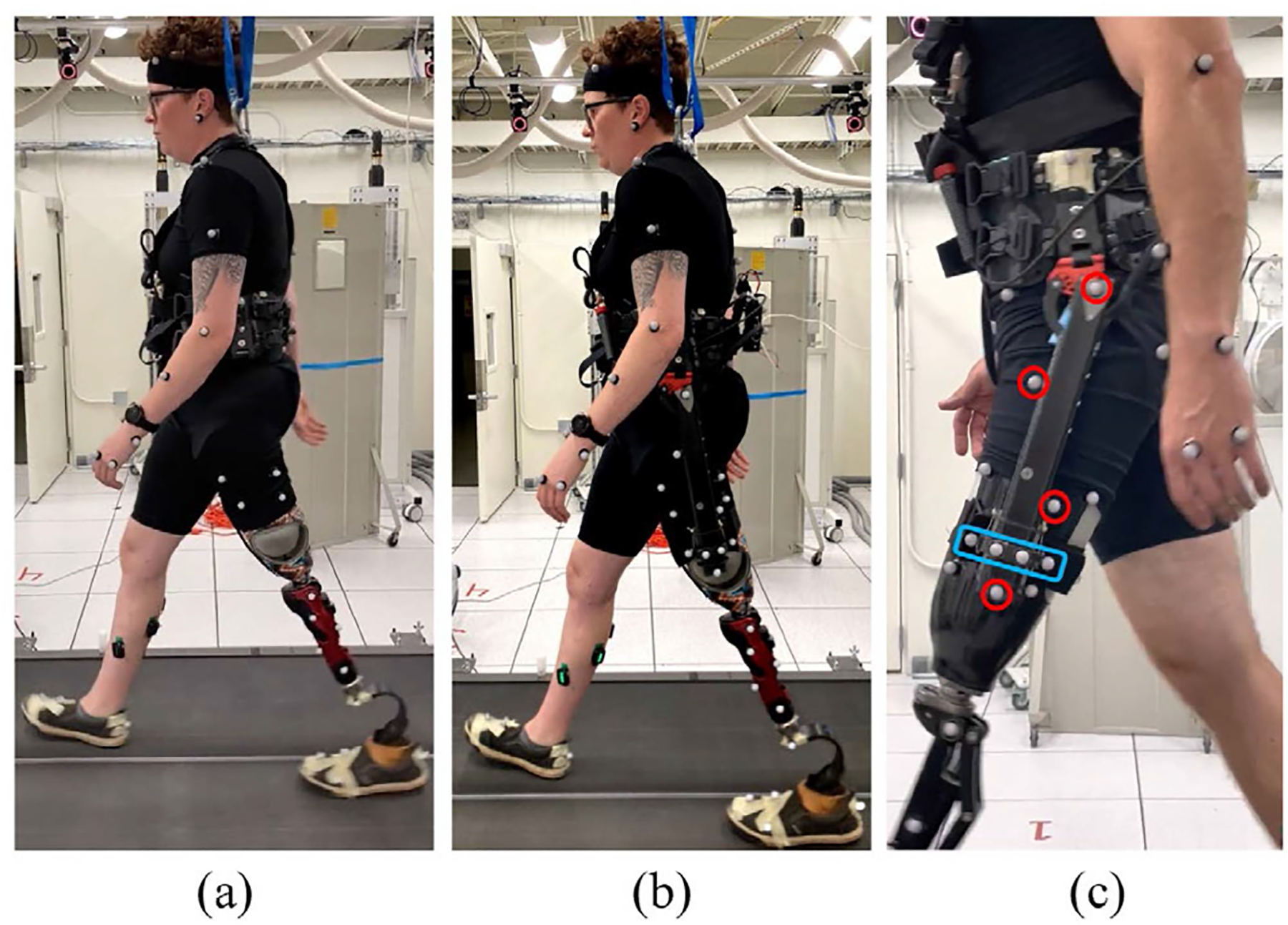
Experimental setup. (a) A subject walks on the instrumented treadmill with reflective markers without the exoskeleton (b) on the instrumented treadmill with reflective markers with the exoskeleton (c) red circles indicate markers on the rigid exoskeleton frame, and the blue rectangle highlights markers on the prismatic passive degree of freedom.

The unilateral, lightweight powered hip exoskeleton used for this study (Fig. 1(a)) was previously presented and validated in healthy and amputee subjects [32]. This exoskeleton provides flexion and extension torque to the user’s residual limb while allowing for passive abduction and adduction. The exoskeleton connects to the user’s socket and includes passive degrees of freedom to reduce the spurious forces and torques on the user’s leg [36], improving comfort [37]. The exoskeleton’s embedded control system runs an assistive controller based on adaptive frequency oscillators [38], [39], [40]. Key parameters of the assistive controller such as the level and timing of the assistance in flexion and extension were tuned for each participant before the start of the experiment. Tuning was based on feedback from the subjects as well as the experimenter’s experience.

### B. Experimental Protocol

We recruited eight participants with unilateral above-knee amputations to participate in this study. Inclusion criteria included age between 18 and 85 years, at least 1 year post amputation surgery, daily use of their prescribed prosthesis, and ability to walk on a treadmill without using handrails. Exclusion criteria included serious comorbidities (including musculoskeletal, cardiac, neuromuscular, skin, or vascular conditions) and inability to communicate or be understood by investigators. All participants had prior experience walking on a treadmill and were considered to be Medicare classification level K3. Information regarding the recruited participants can be found in Table I. The Institutional Review Board at the University of Utah (IRB00099066, approved 07/15/2017) and the U.S. Human Research Protection Office (HRPO) of the U.S. Army Medical Research and Development Command (HSRRB log number: A-19840) approved the study protocol. All participants provided informed consent to participate in the study and to be photographed and video-taped for publication.

Participants wore tight fitting clothing with reflective, cutaneous markers placed on anatomical bony landmarks (Fig. 1). Markers were placed following a modified Plug-in-Gait model with redundant markers placed on the hip, thighs, shins, and feet for more reliable tracking [15]. Additional markers were placed on the pelvis orthosis, which connected the exoskeleton to the user’s waist. As shown in Fig. 1(a), eight markers were placed on the unilateral hip exoskeleton – four to track the prismatic passive degree of freedom and four to track the position and orientation of the actuator relative to the residual limb. Participants wore a harness while walking on the treadmill in case of an adverse walking event.

Calibration of the motion capture model was performed using three routines. Each subject completed a static calibration trial, a functional joint center calibration trial, and a functional dynamic capture. These calibration routines were repeated twice, once without the exoskeleton and once with the exoskeleton. The static calibration created a subject-specific motion capture model. The functional joint center calibration located the centers of rotation for the hip using the Symmetric Center of Rotation Estimation (SCoRE) [34] and knee joint axes using SCoRE and Symmetrical Axis of Rotation Analysis (SARA) [35]. The functional dynamic capture improved the ability of the software to build and label the model for each additional capture. Upon completion of the calibration trials, subjects walked for six minutes on a split-belt treadmill (Bertec, Ohio, USA) at 1 ms^−1^ with instrumented force plates. During the first capture, subjects walked without wearing the exoskeleton, but while wearing the pelvis orthosis to ensure hip markers remained the same between no exoskeleton and exoskeleton conditions. Following the first six-minute capture, subjects donned the unilateral exoskeleton and performed the second set of calibration routines. Subjects walked on the treadmill with the exoskeleton for three six-minute captures to allow subjects to acclimate to the exoskeleton assistance, as in our previous metabolic study [33]. The final fifty strides the no exoskeleton trial and the final fifty strides from the third exoskeleton trial were used for inverse dynamic analysis, as in our previous work [15]. Marker trajectories were sampled at 200 Hz, and ground reaction forces were sampled at 1000 Hz. All data were synchronized during the capture.

### C. Data Analysis

Data were analyzed offline using Nexus (Vicon Motion Systems, Ltd., Oxford, U.K.), Visual 3D (C-Motion, Maryland USA), and MATLAB (MathWorks, Inc. Massachusetts, USA). Marker trajectories and force plate measurements were digitally lowpass filtered using bidirectional, fourth order Butterworth filters at 6 Hz and 15 Hz, respectively. Cutoff frequencies were determined by residual analysis and visual inspection [41]. Ankle joint centers were estimated by markers at the ankle. The SCoRE model was used to calculate the hip kinematics. Both the SCoRE and SARA models were used to calculate the knee kinematics. A variation of the Charnwood Dynamics Model (CODA) pelvis, namely the V3D Composite Pelvis, was used for all hip kinematic analyses. All inverse dynamic calculations were computed using Visual 3D. All data were exported to MATLAB for subsequent analysis.

As is commonly done in the powered exoskeleton field [42], [43], [44], we calculated the biological torque produced by the residual limb of the participants by assuming that the exoskeleton perfectly transfers its desired assistance to the user. Thus, the “biological residual hip torque” was calculated by subtracting the assistive torque provided by the exoskeleton from the “total residual hip torque” calculated through inverse dynamics.

After stride-time normalization, we calculated the average kinematic and kinetic profiles first for each subject across strides, and then calculated the average across the resultant subject means. We also calculated the average of maximum values for each subject across strides, and then calculated the average across the resultant subject means.

Energy was calculated as the time integral of power during each stride. Energetic analyses at the residual hip were divided into subphases as identified in [11]. These phases are identified as H1, H2, and H3, where H1 corresponds to concentric extension power after heel strike, H2 corresponds to eccentric flexion power prior to toe-off, and H3 corresponds to concentric flexion power at toe-off.

To assess changes in center of mass motion, we used the extrapolated center of mass, which relates the lateral center of mass position and the lateral center of mass velocity divided by the subject’s eigenfrequency (defined by the inverted pendulum model). This method has been previously developed and tested on amputee and nonamputee populations [3], [45]. The extrapolated center of mass is useful for assessing stability in dynamic situations [46]. For walking to be considered stable, the extrapolated center of mass should fall between the centers of pressure for the left and right legs. Accordingly, one measure of stability can be calculated by the minimum distance between the average center of pressure of one limb to the peak value of the extrapolated center of mass within a stride.

### D. Statistical Analysis

Statistical analysis was performed only on the primary outcome of the study—the net residual hip energy. Specifically, we performed a paired, two-tailed t-test using MATLAB after verifying normality and homogeneity of variance. We reported 95% confidence intervals (CI) for the no exoskeleton vs exoskeleton conditions for all secondary outcomes, including spatiotemporal, kinematic, and kinetic parameters. These secondary outcomes were analyzed to assess the effect of asymmetric assistance on compensatory behaviors and to verify changes associated with wearing the assistive exoskeleton. Confidence intervals were presented for all secondary outcomes as (lower bound, upper bound).

## III. Results

### A. Residual Hip Effort

**Fig. 2.**
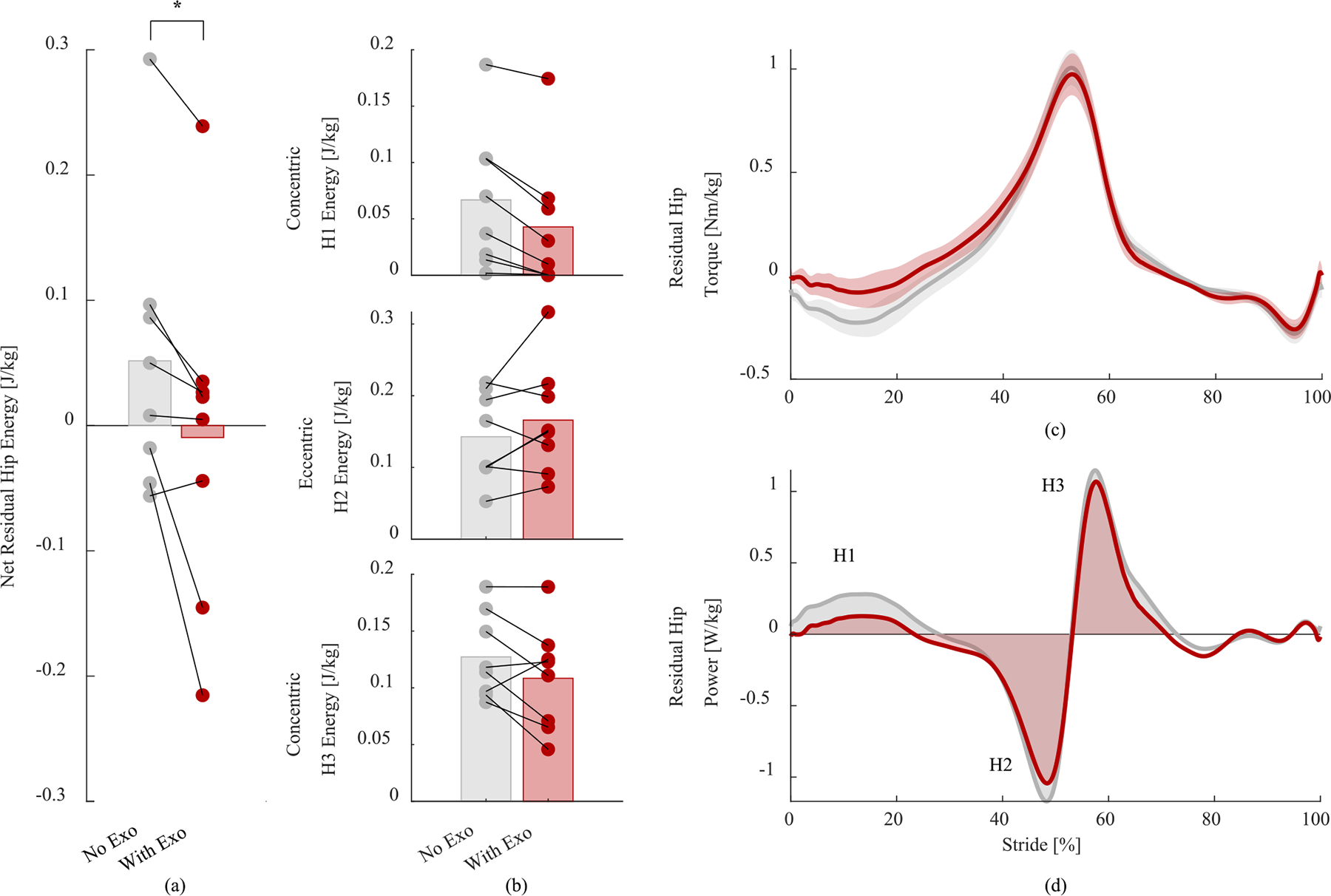
(a) Net residual hip energy (i.e., prosthesis side) averaged across all strides and subjects (bar heights) with individual subject means (circles).Asterisk *indicates statistically significant difference (*p*<0.05) between the “No Exo” and “With Exo” conditions, (b) residual hip energy in concentric and eccentric sub-stride phases, averaged across all strides and subjects (bar heights) with individual subject means (circles), (c) residual hip torque, averaged across all strides and subjects (lines) and standard error of the mean (shading) and (d) residual hip power.). Gray indicates the no exoskeleton condition while red indicates the exoskeleton condition. Bar plots in (b) correspond to the integral of the power phases called out in plot (d).

The net residual hip energy showed a statistically significant reduction while wearing the exoskeleton. Specifically, the net residual hip energy without the exoskeleton was 0.05 ± 0.04 J/kg (mean ± standard error), decreasing to *−*0.01±0.05 J/kg with the exoskeleton (*p* = 0.026, Fig. 2(a)). The observed difference in net residual hip energy resulted from visible changes in both concentric and eccentric energy during different phases of gait. The 95% confidence intervals for all energy and torque are reported in Table II; percent differences are discussed below. On average, with the exoskeleton, concentric energy in the H1 phase decreased by 35.9%, eccentric energy in the H2 phase increased by 14.0%, and concentric energy in the H3 phase decreased by 14.8% (Fig. 2(b), Table II). Also, with the exoskeleton, we observed a 37.5% decrease in biological residual hip extension torque occurring immediately after heel strike, and the peak biological flexion torque decreased by 2.1% (Fig. 2(c), Table II). Similarly, concentric biological power in the residual hip decreased immediately after heel strike (H1 phase) by 35.0% on average (Fig. 2(d)), Table II). Biological peak eccentric power of the residual hip (H2 phase) increased by 13.9% (Fig. 2(d), Table II). Finally, biological peak concentric power of the residual hip (H3 phase) decreased slightly by 3.0% (Fig. 2(d), Table II).

**Fig. 3.**
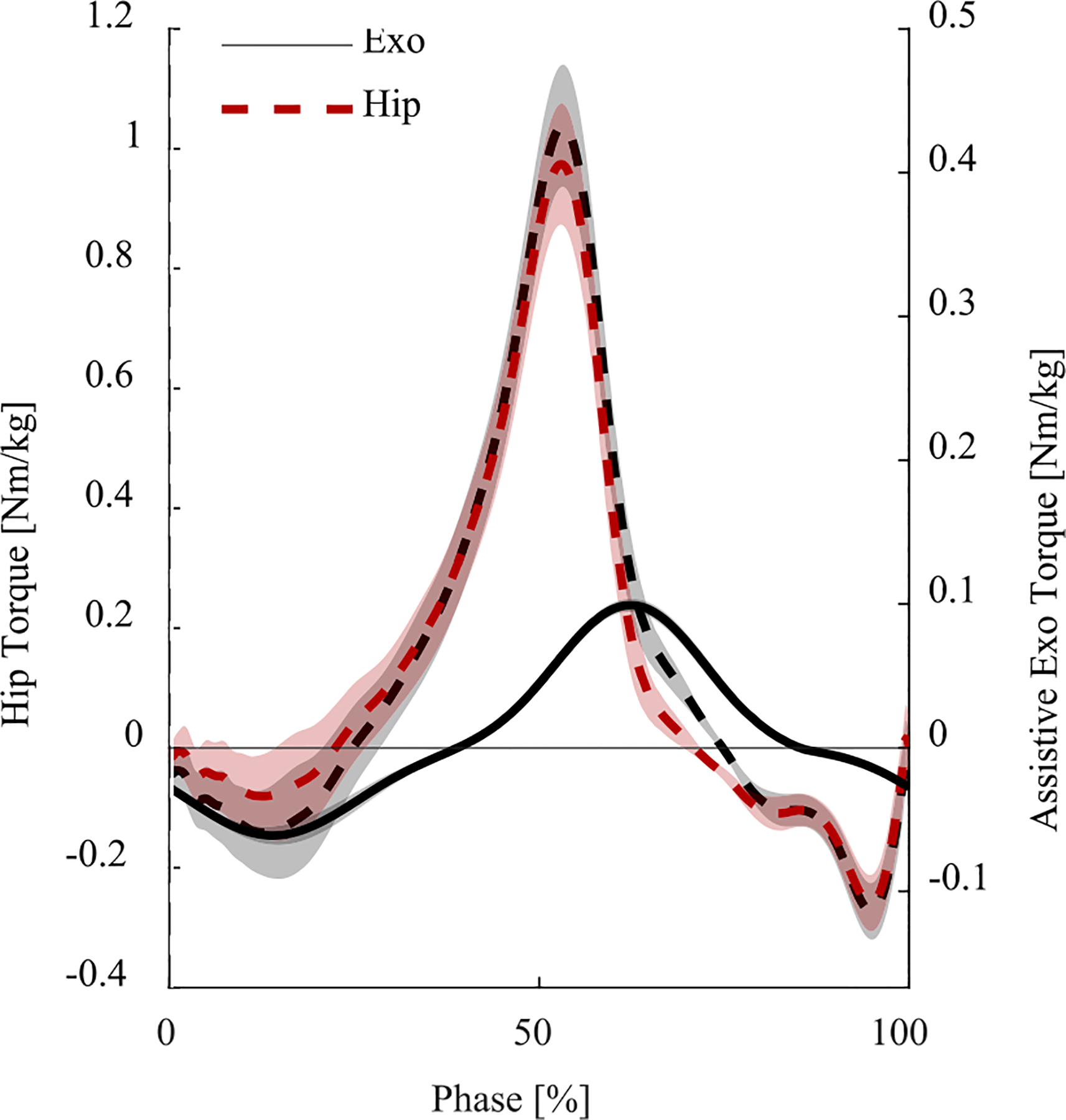
Prosthesis side hip torques during the exoskeleton trial, averaged across all strides and subjects, with shading representing standard error of means. The left y-axis shows the total residual hip torque (including exoskeleton torque) (dashed black line) and the biological residual hip torque (no exoskeleton torque) (dashed red line). The right y-axis shows the assistive torque applied by the exoskeleton (solid black line).

On average, the exoskeleton provided 0.10 ± 0.01 Nm/kg of flexion assistance, peaking at 62.4% stride. Also, on average, the exoskeleton provided 0.06 ± 0.01 Nm/kg of extension assistance, peaking at 13.9% stride (Fig. 3). Fig. 3 shows the mean total residual hip torque profile calculated using inverse dynamics (black dashed line) superimposed on the mean assistive torque profile provided by the exoskeleton (black, solid line) and the mean biological torque profile of the residual hip (red, dashed line).

### B. Center of Mass, Center of Pressure, and Kinematics

**Fig. 4.**
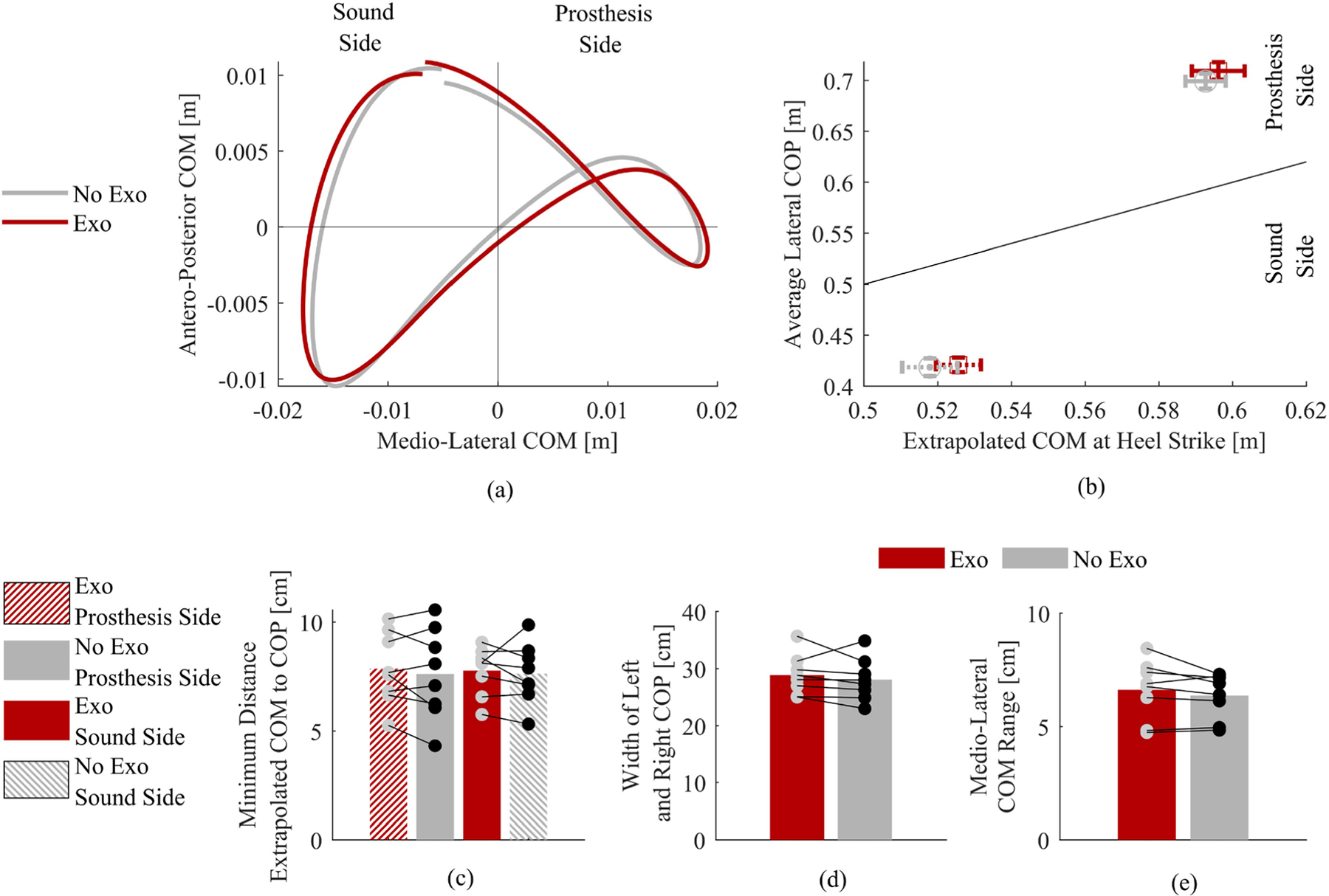
Secondary analysis of balance and stability. (a) Center of mass in the medial-lateral direction and the anterior-posterior direction, averaged across all strides and subjects. (b) Center of pressure averaged across all strides and subjects, plotted against the extrapolated center of mass position at heel strike averaged across all strides and subjects. Error bars represent standard error of means. (c) Minimum distance from the center of mass to the extrapolated center of mass for each side and condition. (d) Average distance between the sound side and prosthesis side center of pressure (COP) between exoskeleton and no exoskeleton conditions. (e) Total medial-lateral range of motion while wearing the exoskeleton compared to no exoskeleton. (c)–(e) Display the means across all strides and subjects (bar heights) and individual subject means (circles).

The trajectory of the medio-lateral and antero-posterior center of mass (COM) was not substantially affected by the exoskeleton assistance (Fig. 4(a)). Only small differences were observed in the lateral center of pressure (COP) between the exoskeleton condition (1.0 cm more lateral, prosthesis side; 0.2 cm more lateral, sound side) compared to the no exoskeleton condition (Fig. 4(b)). Similarly, the extrapolated COM at heel strike was 0.34 cm more lateral on the prosthesis side and 0.78 cm more lateral on the sound side when using the exoskeleton compared to without it (Fig. 4(b)). Fig. 4 also shows the minimum distance between extrapolated COM and average COP (c), the average distance between left and right COP (d), and the average medio-lateral center of mass range (e). Only small differences, on the order of 4%, were observed in COM- or COP-related measures between tested conditions. Table III shows 95% confidence intervals for COM and COP with and without the exoskeleton.

**Fig. 5.**
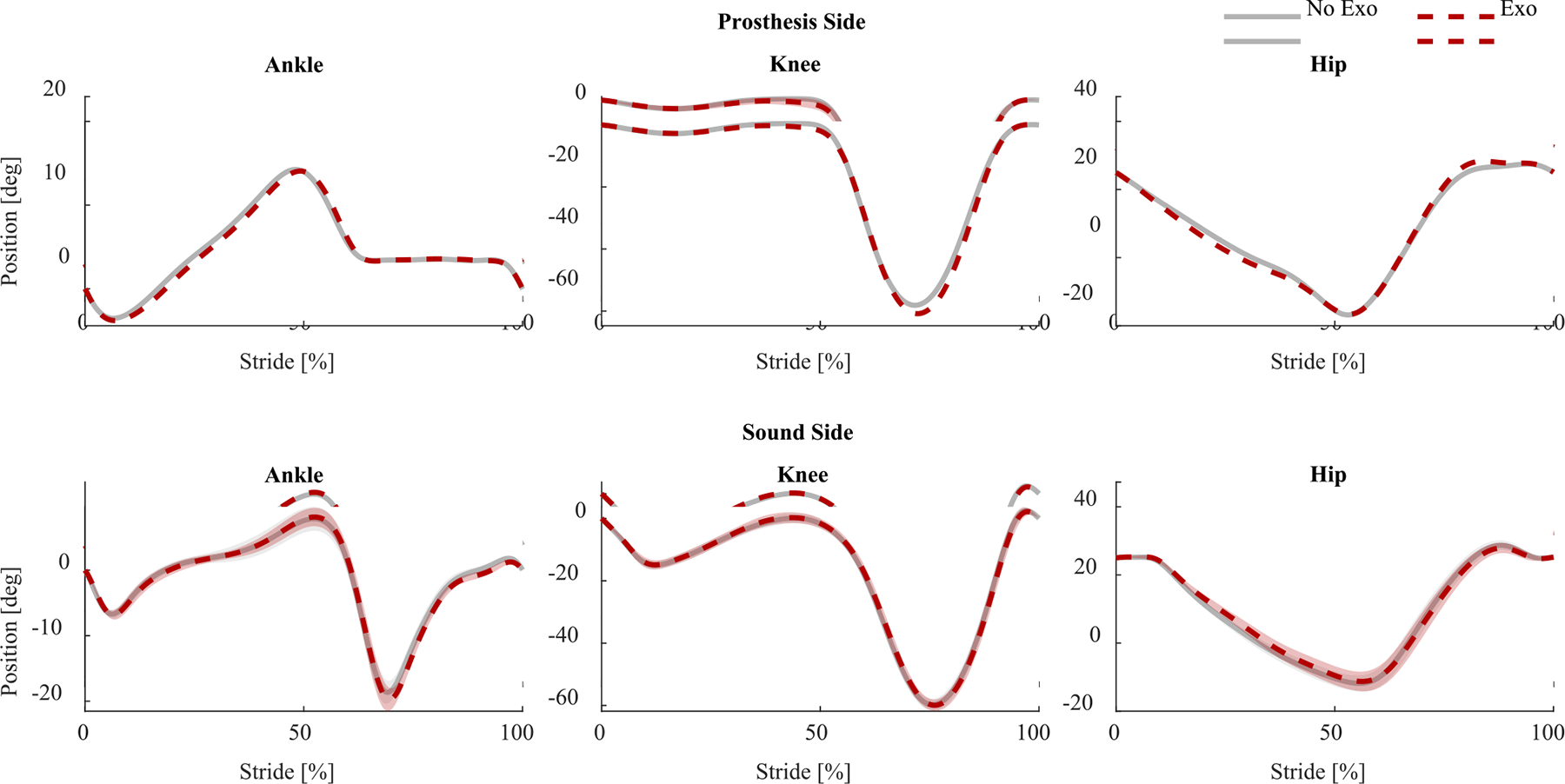
Ankle, knee, and hip kinematics for the prosthesis side (top row) and sound side (bottom row) during the exoskeleton (dashed red line) and no exoskeleton (solid grey line) conditions, averaged across all strides and subjects. Shaded areas represent the standard error of means.

The kinematic profiles for both the prosthesis side and the sound side, with and without the exoskeleton, are shown in Fig. 5. We reported the 95% confidence intervals for all kinematic variables in Table III. Similar ranges of motion (ROM) for all joints were observed with and without the exoskeleton, and differences between conditions were lower than 4.5%. Peak extension velocity of the residual hip increased by 6.3% with the exoskeleton. Moreover, peak extension velocity of the sound side hip decreased by 4.7% with the exoskeleton. Peak flexion velocity of the prosthesis side knee increased by 6.1% with the exoskeleton. Peak flexion velocity of the sound side knee was minimally affected by the exoskeleton. Peak flexion and extension velocities of both prosthesis and sound ankles were not visibly affected by the exoskeleton.

### C. Spatiotemporal Analysis

Spatiotemporal parameters for both the prosthesis side and sound side were mostly unaffected by the exoskeleton assistance. Observed differences in spatiotemporal parameters were lower than 3%, as seen in Table IV, where we report the 95% confidence intervals for all spatiotemporal parameters for both the prosthesis side and sound side, with and without the exoskeleton.

## IV. Discussion

Powered hip exoskeletons have shown the ability to improve meaningful clinical outcomes for individuals with above-knee amputations, including walking economy [33], stumble recovery [31], and self-selected walking speed after exoskeleton mediated training [30]. However, previous studies have not provided a mechanistic explanation for how the assistance provided by a powered hip exoskeleton affects amputee gait biomechanics during walking. This study tested the hypothesis that the assistance provided by the hip exoskeleton decreases the torque generated by the residual limb muscles of the hip joint, reducing the residual limb effort. Our experiments with eight subjects with above-knee amputations supported this hypothesis by showing that the net residual hip energy, calculated as the integral of the biological hip power over the stride duration, significantly decreased by 80% when walking with the exoskeleton, compared to walking without the exoskeleton. The analysis of the hip energy during different gait phases showed that the powered hip exoskeleton had a more noticeable effect on the concentric residual hip energy than on the eccentric residual hip energy. This analysis suggests that the observed reduction in net residual hip energy is mainly due to reduction in the concentric energy generated by the residual limb muscles.

This study suggests that the reduction in net residual hip energy is a major factor in explaining the walking economy improvements previously observed [33]. We believe that the reduction in net residual hip energy offers a plausible mechanism to explain observed metabolic improvements, because the hip joint has been shown to have low efficiency in converting metabolic to mechanical energy in healthy individuals [47]. We also expect that the residual hip joint in above-knee amputee individuals has a much lower efficiency than in nonamputees, given the substantial shortening and non-physiological arrangement of the residual hip muscles after amputation [10]. Therefore, reducing the mechanical effort at the hip could have a substantial impact on metabolic energy during walking.

The analysis of subject-specific results provided additional insights into how the hip mechanics changed with the exoskeleton assistance. While the average net residual hip energy decreased significantly, achieving close to zero sample mean, for some subjects the magnitude of net residual hip energy increased – for these subjects, the net residual hip energy was negative without the exoskeleton, and became more negative with the exoskeleton. Secondary analysis showed that, for these subjects, the net residual hip energy became more negative because of a concurrent reduction of the positive concentric hip energy (H1 and H3) and increase (in magnitude) of the negative eccentric hip energy (H2). We believe that this result agrees with the metabolic cost reduction observed in our previous study because positive energy generation has higher metabolic cost than negative energy absorption at the muscle level [48].

The powered hip exoskeleton reduced the peak hip extension torque of the residual limb during early stance by 37.5% (Fig. 2(c)). The reduction in hip extension torque could be particularly helpful because the residual hip extensors in individuals with above-knee amputations are generally weak [11], [49]. Interestingly, there were no visible differences in residual hip peak flexion torque despite the residual hip flexors receiving substantial assistance from the exoskeleton. This result may be due to the timing of the hip flexion assistance, which seems to be slightly late compared to the torque produced by the residual limb (Fig. 3). Thus, although the powered hip exoskeleton assisted the residual limb both in flexion and extension, the extension assistance seems to be the main contributor to the observed reduction in residual limb energy and effort. Further tests are necessary to assess the effect of timing and magnitude of assistance in flexion and extension on biomechanical adaptations.

In this study, we chose to assist only the residual limb using an asymmetric, unilateral exoskeleton configuration. Choosing to assist the residual limb might have further exacerbated existing kinematic and kinetic compensatory strategies or affected COM and COP trajectories. However, there were no meaningful changes to the antero-posterior or medio-lateral motion of the COM when participants used the exoskeleton (Fig. 4). Compared to nonamputees, above-knee amputees walk with a wider center of pressure between limbs [3]. We observed no meaningful changes in the COP of either limb relative to the extrapolated COM while walking (Fig. 4(b)). Thus, the analysis of the center of mass (COM) and center of pressure (COP) suggests that the exoskeleton assistance does not have any apparent effects on balance. The kinematic analysis shows only minor differences between walking with and without the exoskeleton. Also, there were no visible changes in ROM on either limb, which does not support our previous study in which a significant increase in the ROM of the residual limb was reported [33]. Although no changes in joint angles were observed, we did identify significant changes in velocity. The residual hip extension velocity increased by 6.3% and the sound hip extension velocity decreased by 4.7% when wearing the exoskeleton. Although residual hip extension velocity increased, no corresponding increase in concentric extension power was observed. In fact, peak concentric residual hip extension power decreased due to the decrease in extension torque. This secondary kinematic analysis provides interesting insights into the adaptation mechanisms observed during the powered hip exoskeleton assistance. However, the observed changes are generally small and, therefore, do not seem to play a major role in explaining how above-knee amputees adapt to powered hip exoskeletons.

The effects of the powered hip exoskeleton on spatiotemporal parameters and gait symmetry were minimal and unlikely to be clinically meaningful. The study participants showed relatively symmetric gait (Table IV, Fig. 4, Fig. 5). This result is not surprising given that the inclusion criteria required subjects to be able to walk on a treadmill without handrails, which is only possible for above-knee amputees with a high level of mobility. On the other hand, based on the observed changes in joint velocities, we would have expected to see more substantial changes in stance time, swing time, or step lengths.

### A. Limitations

A limitation of this study is that we were not able to include electromyography. Understanding the influence of the exoskeleton on muscle activations would have added important insights into the mechanisms used to adapt to the exoskeleton assistance. Notably, we did not observe any meaningful changes in COM kinematics, which would likely be linked to changes in lower-back muscle activity. Also, we observed a reduction in residual limb extension torque, which could indicate reduced effort in the extensor muscles in the residual limb. Future work should explore the changes in lower-limb muscular effort while wearing the hip exoskeleton, similar to what was recently done in nonamputee individuals [50].

Another limitation of this study is that we assumed a perfect transmission of torque from the exoskeleton to the user’s residual hip. Although this is a common assumption made in the field, it can have an impact on the interpretation of the results. In a previous study [41], we used a 6-axis loadcell at the interface between a powered knee exoskeleton and the user to directly measure how the torque was transmitted from the exoskeleton to the user. The result of this study suggested that the assumption of perfect transfer of torque is reasonable provided that a self-aligning mechanism is implemented in the exoskeleton. Self-aligning mechanisms can significantly reduce spurious forces and torques between the user and the exoskeleton interface, therefore minimizing inefficiencies [41]. They also have a significant effect on performance and comfort [42]. Because the powered hip exoskeleton used in this study uses a self-aligning mechanism [51], the assumption of perfect transmission of forces and torques to the user should not cause substantial inaccuracies.

Participants with lower mobility or higher asymmetry may respond to the residual hip assistance provided by the powered hip exoskeleton in a different way compared to what we observed in this study. In the present study, participants were required to walk on a treadmill continuously without using the handrails for assistance. Consequently, the participants were of a high mobility index, which limits our understanding of how the exoskeleton might change kinematics and kinetics of individuals with a low mobility index. Future work should assess the impact of unilateral hip assistance in a lower mobility population, because the hip exoskeleton might prove even more beneficial to lower mobility populations.

Another limitation of this study is that we did not use any subjective measures of effort, such as rated perceived exertion. Our study participants subjectively commented on the assistance from the hip exoskeleton, likening it to using an electric bicycle. In fact, many participants commented on the difficulty of walking after doffing the exoskeleton. These subjective remarks are encouraging, but do not offer qualitative information regarding exoskeleton assistance outside of laboratory environments. Future work should include subjective analyses regarding user preferences and real-world applications.

Finally, understanding how assistive devices interact with prescribed passive prostheses is an important step in making powered exoskeletons a clinically viable intervention. Changing user prosthesis alignment might influence the interaction between the prosthesis and exoskeleton. Prosthesis tuning was not explored as a variable in this study and represents a limitation.

## V. Conclusion

In this paper, we evaluated the effect of a unilateral, lightweight powered hip exoskeleton on the gait biomechanics in eight above-knee amputees. The powered exoskeleton assistance significantly reduced the net residual hip energy, with the concentric H1 energy (0–30% gait cycle) being the main contributor to this change. Moreover, the powered exoskeleton assistance visibly affected the residual hip extension torque. Notably, the powered exoskeleton assistance had negligible effects on joint angles, base of support, and centers of pressure. This result indicates that the powered exoskeleton did not negatively affect balance and stability. Future work should aim to improve balance and stability, which are typically poor in individuals with above-knee amputations. We believe that this outcome could be achieved by assisting the residual hip joint in abduction and adduction to provide frontal plane stability and assistance. Future work should also aim to assess long-term efficacy in a take-home trial or understand the importance of an assistive profile based on the needs of the population.

## Figures and Tables

**TABLE I:** Participant Demographics

Subject	Age [yrs]	Weight [kg]	Height [m]	Sex	Years since Amputation	Knee Prosthesis	Ankle Prosthesis	Amputation Side	Socket Suspension
S1	73	79.5	1.65	M	5	C-Leg	Triton	L	Lanyard
S2	52	109.1	1.73	M	4	Genium X3	Triton	R	Lanyard
S3	37	100.5	1.80	M	9	C-Leg	All Pro	L	Suction
S4	28	65.1	1.78	M	7	Plie	All Pro	R	Suction
S5	31	77.3	1.80	M	3	KX06	Hi Pro	L	Suction
S6	31	59.1	1.60	F	12	Plie	All Pro	L	Lanyard
S7	51	102.3	1.91	M	9	C-Leg	Triton	L	Pin Lock
S8	39	90.5	1.91	M	35	Plie	Soleus	L	Suction

**TABLE II:** 95% Confidence Intervals for Residual Hip Joint Energy, Power, and Torque

		Exo	No Exo
HI	Energy (J/kg)	(0.03, 0.05)	(0.06, 0.08)
	Power (W/kg)	(0.19,0.33)	(0.34, 0.46)
H2	Energy (J/kg)	(−0.15, −0.19)	(−0.13, −0.15)
	Power (W/kg)	(−1.09, −1.27)	(−1.28, −1.46)
H3	Energy (J/kg)	(0.10, 0.12)	(0.12, 0.14)
	Power (W/kg)	(1.26, 1.50)	(1.33, 1.53)
Peak Extension Torque After Heel Strike [Nm/kg]		(0.17, 0.23)	(0.28, 0.36)
Peak Flexion Torque [Nm/kg]		(0.86, 1.22)	(0.90, 1.22)

**TABLE III:** Confidence Intervals of Center of Mass (COM), Center of Pressure (COP), Range of Motion (ROM), and Joint Velocity for Sound Side and Prosthesis Side

		Prosthesis Side	Sound Side
Peak Medio-Lateral COM [cm]	Exo	(6.3, 6.9)	
	No Exo	(6.1, 6.6)	
Minimum Distance from Extrapolated COM to COP [cm]	Exo	(6.9, 7.7)	(7.7, 8.3)
	No Exo	(6.5, 7.5)	(7.4, 8.2)
Ankle ROM [°]	Exo	(14.3, 22.9)	(26.9, 33.5)
	No Exo	(14.5 22.4)	(26.5,31.3)
Knee ROM [°]	Exo	(56.4 70.9)	(61.7, 69.2)
	No Exo	(53.9, 68.3)	(61.7, 69.0)
Hip ROM [°]	Exo	(42.8, 50.8)	(37.0, 46.5)
	No Exo	(42.7, 49.0)	(38.4, 47.2)
Peak Flexion Knee Velocity [°/s]	Exo	(385.7, 410.3)	(399.6, 465.0)
	No Exo	(363.3, 386.7)	(395.5, 463.3)
Peak Extension Hip Velocity [°/s]	Exo	(196.2, 207.8)	(177.2, 188.8)
	No Exo	(183.7, 196.3)	(185.6, 198.4)

**TABLE IV:** Confidence Intervals of Spatiotemporal Parameters

		Prosthesis Side	Sound Side
Stride Time [s]	Exo	(1.16, 1.18)	(1.16, 1.18)
	No Exo	(1.16, 1.18)	(1.16, 1.18)
Stance Time [s]	Exo	(0.72, 0.74)	(0.79, 0.80)
	No Exo	(0.70, 0.72)	(0.79, 0.80)
Swing Time [s]	Exo	(0.44, 0.46)	(0.36, 0.38)
	No Exo	(0.45, 0.47)	(0.36, 0.38)
Cadence [steps/min]	Exo	(94.8, 97.0)	(108.3, 111.7)
	No Exo	(94.9, 97.3)	(108.5, 112.1)
Step Length [m]	Exo	(0.74, 0.76)	(0.55, 0.61)
	No Exo	(0.74, 0.76)	(0.60, 0.62)
Step Width [m]	Exo	(0.23, 0.24)	
	No Exo	(0.23, 0.24)	
Minimum Prosthesis Side Toe Clearance [cm]	Exo	(7.00, 7.66)	
	No Exo	(6.95, 7.35)	
